# STIL Acts as an Oncogenetic Driver in a Primary Cilia-Dependent Manner in Human Cancer

**DOI:** 10.3389/fcell.2022.804419

**Published:** 2022-01-26

**Authors:** Jingxian Li, Zikun Yang, Yuanjiong Qi, Xun Liu, Yang Liu, Xinyu Gao, Shuai Li, Jianqiang Zhu, Changwen Zhang, E Du, Zhihong Zhang

**Affiliations:** ^1^ Tianjin Institute of Urology, The Second Hospital of Tianjin Medical University, Tianjin, China; ^2^ Department of Graduate School, Tianjin Medical University, Tianjin, China

**Keywords:** primary cilia, pan-cancer, STIL, Shh signaling, cell cycle

## Abstract

SCL/TAL1 Interrupting locus (STIL) is a ciliary-related gene involved in regulating the cell cycle and duplication of centrioles in dividing cells. STIL has been found disordered in multiple cancers and driven carcinogenesis. However, the molecular mechanisms and biological functions of STIL in cancers remain ambiguous. Here, we systematically analyzed the genetic alterations, molecular mechanisms, and clinical relevance of STIL across >10,000 samples representing 33 cancer types in The Cancer Genome Atlas (TCGA) dataset. We found that STIL expression is up-regulated in most cancer types compared with their adjacent normal tissues. The expression dysregulation of STIL was affected by copy number variation, mutation, and DNA methylation. High STIL expression was associated with worse outcomes and promoted the progression of cancers. Gene Ontology (GO) enrichment analysis and Gene Set Variation Analysis (GSVA) further revealed that STIL is involved in cell cycle progression, Mitotic spindle, G2M checkpoint, and E2F targets pathways across cancer types. STIL expression was negatively correlated with multiple genes taking part in ciliogenesis and was positively correlated with several genes which participated with centrosomal duplication or cilia degradation. Moreover, STIL silencing could promote primary cilia formation and inhibit cell cycle protein expression in prostate and kidney cancer cell lines. The phenotype and protein expression alteration due to STIL silencing could be reversed by IFT88 silencing in cancer cells. These results revealed that STIL could regulate the cell cycle through primary cilia in tumor cells. In summary, our results revealed the importance of STIL in cancers. Targeting STIL might be a novel therapeutic approach for cancers.

## Introduction

The primary cilia (PC) is a microtubule-based structure that protrudes from the surface of almost all mammalian cells and transduces extracellular signals to the cell body to regulate diverse cellular, developmental and physiological processes (Gluenz et al., 2010). Studies have previously shown a loss of primary cilia in multiple cancer types (Seeley et al., 2009; Basten et al., 2013; Nobutani et al., 2014; Du et al., 2018; Qie et al., 2020), revealing that the repression of primary cilia formation is closely related to carcinogenesis. SCL/TAL1 Interrupting locus (STIL) is a primary cilia-regulated gene (Reiter and Leroux, 2017). STIL expression balance is closely involved in the formation of primary cilia (Patwardhan et al., 2018). Moreover, STIL is a cell-cycle-regulated protein specifically recruited at the mitotic centrosome to promote the duplication of centrioles in dividing cells. Complete loss of STIL expression results in no primary cilia. In several cancers with bad prognosis, STIL expression has been found up-regulation, including lung cancer (Goto et al., 2013), prostate adenocarcinoma (Wu et al., 2019), bladder cancer (Du et al., 2018), and ovarian cancers (Rabinowicz et al., 2017a). Moreover, STIL over-expression is correlated with increased metastatic potential in cancers (Ramaswamy et al., 2003). However, even though in tumors with up-regulated STIL expression, the number of primary cilia still declines (Seeley et al., 2009; Basten et al., 2013; Nobutani et al., 2014; Du et al., 2018; Qie et al., 2020). Therefore, the role of STIL for primary cilia formation still needs to explore in the tumor.

Our study systematically explored the genetic alterations, molecular mechanisms, and clinical relevance of STIL across cancer types. STIL was widely up-regulated in multiple cancer types compared with adjacent normal tissues. Meanwhile, STIL over-expression was related to the progression of cancers and influenced the prognosis of cancer patients. We further analyzed the reasons leading to the disturbance of STIL expression and found that STIL expression was subjected to mutation, DNA methylation, and copy number variation (CNV). GO enrichment analysis revealed that STIL was involved in the biological process associated with the cell cycle, which was consistent with the previous study (Arquint et al., 2012; Patwardhan et al., 2018). Moreover, STIL expression was highly correlated with several hall-mark cancer pathways, including Mitotic spindle, G2M checkpoint, and E2F targets pathways across 33 cancer types, suggesting STIL played a stable molecular function in pan-cancer. Finally, we validated that STIL participated in the primary cilia formation and regulated cell cycle progression in prostate cancer and kidney cancer cell lines, revealing that STIL played a critical role in regulating cancer cells through primary cilia. As a potentially pivotal gene, STIL might be a novel therapeutic target for cancers.

## Results

### The Expression Level and Clinical Relevance of STIL

**FIGURE 1:**
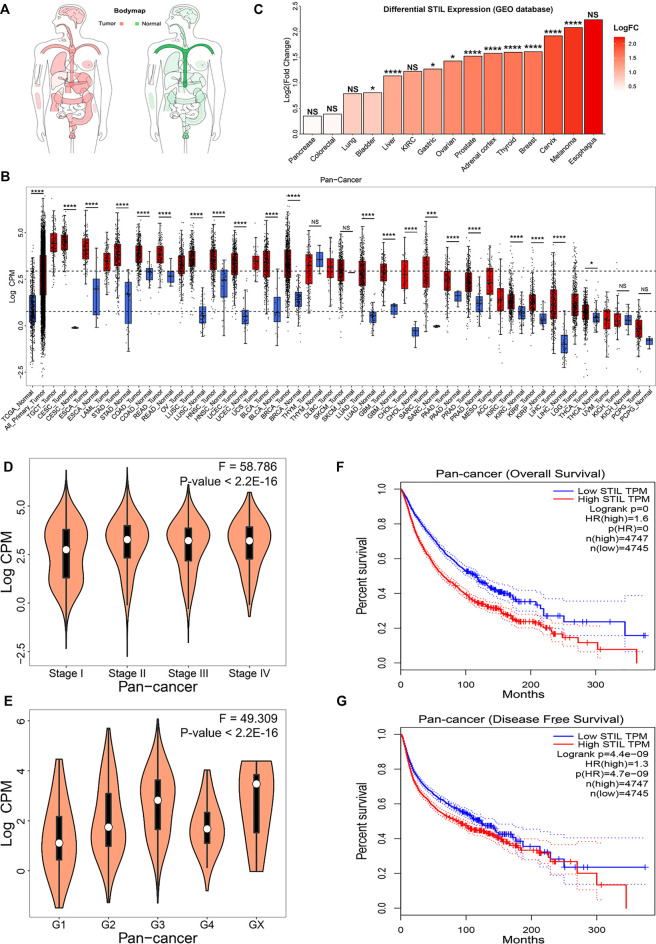
STIL mRNA expression in cancer and normal tissues and clinical relevance. **(A)** The body map depicted by the GEPIA website showed the distribution of STIL expression levels in humans. **(B)** STIL expression levels in the TCGA normal and TCGA cancer tissues. Sample lines represent medians and quartiles of STIL expression. Low and high dashed lines across the graph represent STIL median expression for TCGA normal and TCGA cancer, respectively. *p*-values are identified with an asterisk. **(C)** STIL expression alteration comparing tumor tissue with normal tissue in different cancer types for GEO datasets. *p*-values are identified with an asterisk. **(D)** The violin diagram depicted the STIL mRNA expression in the different pathological stages in pan-cancer. **(E)** The violin diagram depicted the STIL mRNA expression in different histologic grades in pan-cancer. Kaplan-Meier survival curves of patients’ OS **(F)** and RFS **(G)** grouped by the global expression pattern of STIL in pan-cancer. The log-rank test *p*-values are shown. *p-value < 0.05; **p-value < 0.01; ***p-value < 0.001; ****p-value < 0.0001; NS, No Significance.

The body map depicted by the GEPIA website showed that STIL was widely expressed in human tissues (Figure 1A), which demonstrated that STIL is suitable for a pan-cancer analysis. To explore the expression level of STIL, we obtained the mRNA expression profiling of STIL from the TCGA and GEO datasets (Supplementary Table S1). We portrayed a STIL pan-cancer expression landscape (Figure 1B) using the TCGA dataset. We plotted two dashed lines in this landscape. The upper dashed line represents the median expression level of STIL in all TCGA primary tumor tissues. The lower dashed line represents the median expression level of STIL in the TCGA normal tissues. We found STIL expression was higher in most cancer types compared with the STIL expression in all normal tissues. Moreover, STIL was upregulated in multiple primary tumor types (including the CESC, ESCA, STAD, COAD, READ, LUSC, HNSC, UCEC, BLCA, BRCA, LUAD, GBM, CHOL, SARC, PRAD, KIRC, KIRP, THCA, and LIHC) comparing with their corresponding adjacent normal tissues. There were few or no corresponding normal tissues for TGCT, LAML, OV, UCS, SKCM, and MESO in the TCGA dataset, but STIL showed higher expression in these cancers than the median STIL expression of all TCGA normal tissues. In THYM, PAAD, KICH, and PCPG, STIL showed no expression alteration compared with their corresponding normal tissues. The GEO datasets were further analyzed to verify the STIL expression alteration in cancers. Through a differential expression analysis, we found STIL was also upregulated in multiple cancer tissues, including bladder, liver, gastric, ovarian, prostate, adrenal cortex, thyroid, breast, cervix, and skin cancer, compared with their normal tissues (Figure 1C), which was consistent with the results of TCGA dataset.

To explore the relationship between tumor progression, prognosis, and STIL expression, we collected the clinical data from the TCGA database. We found that STIL expression was elevated, accompanied by the advanced pathological stage and histologic grade in pan-cancer level (Figures 1D,E). In individual cancer (including ACC, BRCA, KICH, KIRP, LIHC, LUAD, and PAAD), STIL expression also increased with advanced pathological stage, suggesting that STIL may promote the progression of these cancers (Supplementary Figure S1). Furthermore, the patients with high STIL expression showed worse outcomes in pan-cancer (Figures 1F,G). In multiple cancers (including ACC, BLCA, KICH, KIRC, KIRP, LGG, LIHC, LUAD, MESO, PAAD, PRAD, SARC, PCPG, THCA, UCEC, and UVM), an increase of STIL expression was a risky factor for overall survival (OS) or recurrence-free survival (RFS) of cancer patients (Supplementary Table S2). The patients with high STIL expression showed worse clinical outcomes and promoted cancer recurrence (Supplementary Figures S2, S3). Nevertheless, in READ and THYM, high STIL expression may prevent patients from death and tumor recurrence. Consistent results were also found in GEO datasets, in BLCA, Astrocytoma, SKCM, LUAD, BRCA, and Glioma, patients with high STIL expression showed worse prognosis (Supplementary Figure S4). In contrast, high STIL expression patients showed a better prognosis in colorectal cancer.

### The Genetic Alterations and Aneuploidy Relevance of STIL Across Cancer Types

**FIGURE 2:**
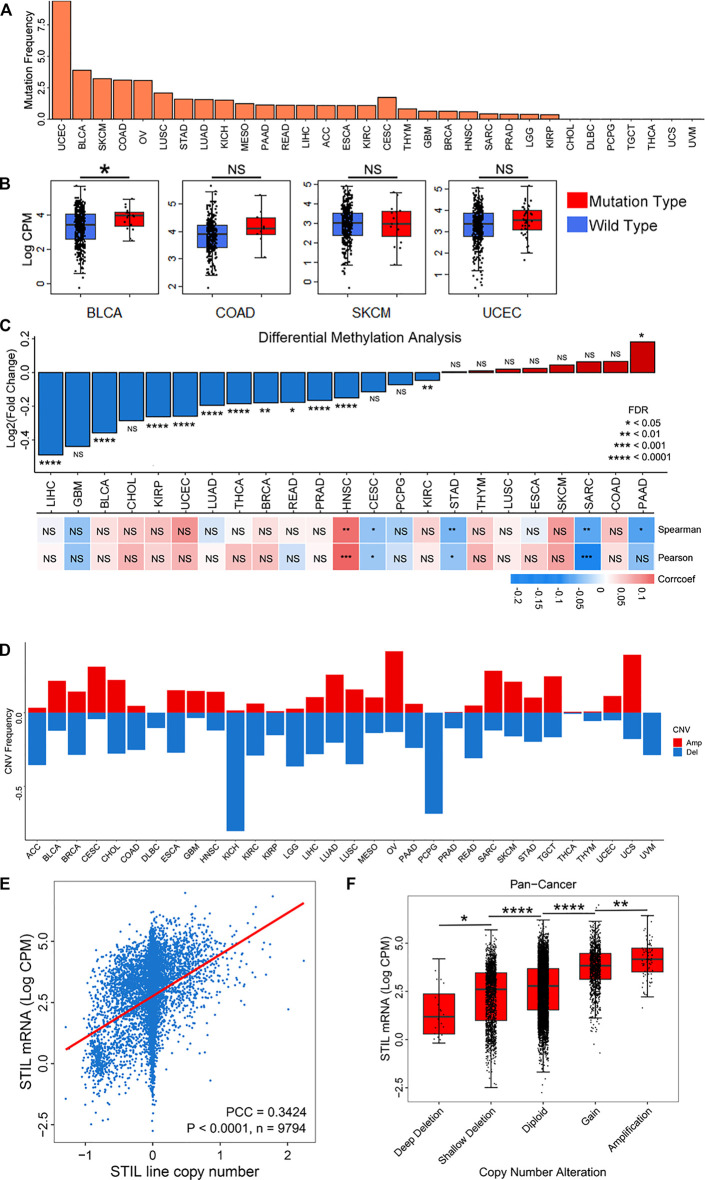
Mutation, DNA methylation, and CNV characterization of STIL in cancers. **(A)** The mutation frequency of STIL across 32 cancer types. **(B)** STIL expression for wild types and mutation types in BLCA, COAD, SKCM, and UCEC. **(C)** STIL DNA methylation alterations (Beta values) comparing tumor tissues with normal tissues. Red represent Log_2_(Fold change) > 0, blue represents Log_2_(Fold change) < 0. Pearson and Spearman correlation coefficients were calculated to show the correlation between STIL mRNA expression and DNA methylation beta values. A red box showed a positive correlation, and a blue box showed a negative correlation. **(D)** Amplification and deletion frequency of STIL across 32 cancer types. The red rectangle showed copy number amplification, and the blue rectangle represents copy number deletion. **(E)** Spearman correlation coefficient between STIL mRNA expression and copy number variation in pan-cancer levels. *p*-value, sample number, and Spearman r are shown in the box. **(F)** STIL mRNA expression in different copy number variation situations in pan-cancer levels. *p*-value and sample number are shown in the box. *p-value < 0.05; **p-value < 0.01; ***p-value < 0.001; ****p-value < 0.0001; NS, No Significance.

To explore the reasons for STIL expression alteration, we analyzed the epigenetic alteration of STIL, including mutation, DNA methylation, and copy number variation. STIL mutation characterization was first paid attention (Figure 2A). We found STIL mutated in most cancers, but the mutation frequency was generally low, ranging from 0% to 9.4%. In UCEC, BLCA, SKCM, OV, and COAD, the mutation rate of STIL (>2.5%) were higher than that in other cancer types. UCEC, BLCA, SKCM, and COAD were selected for subsequent analysis to explore the effect of mutation on expression. Ovarian cancer was excluded because the samples were small and STIL only mutated in two of these samples. In BLCA, STIL expression was higher in mutation types than that in wild types. However, STIL expression showed no significant difference in mutation and wild types in the other three cancers (Figure 2B). We then analyzed the correlation between DNA methylation and STIL expression across cancer types (Figure 2C, Supplementary Table S3). The methylation beta value was significantly down-regulated in multiple cancers (|LogFC| ≥ 0.2, *p*-value < 0.05), including LIHC, BLCA, KIRP, and UCEC. In these four cancer types, STIL DNA methylation showed no correlation with STIL expression. In CESC, STAD, SARC, and PAAD, DNA methylation showed a negative correlation with STIL expression. In HNSC, DNA methylation showed a positive correlation with STIL expression. (Figure 2C; Supplementary Table S3). However, these correlations were not significant. Finally, we focused on the STIL copy number variation (CNV) (Figure 2D). STIL showed a high amplification ratio in TGCT, CESC, OV, UCS, SARC, and BLCA, representing higher expression in the pan-cancer expression landscape (Figure 1B). In contrast, STIL showed a high deletion ratio accompanied by lower expression in ACC, KIRC, KIRP, PCPG, LGG, and UVM. In pan-cancer levels, STIL copy number represented a positive correlation with STIL mRNA expression (Figure 2E). Meanwhile, We observed a progressive increase in STIL mRNA with increased copy number (Figure 2F). The similar results between STIL mRNA expression and CNV were also observed across pan-cancer cell lines from the CCLE dataset (Supplementary Figures S5A and S5B), suggesting that STIL CNV also played an important role in regulating STIL expression level.

STIL overexpression causes centrosome overreplication and increases the opportunity of aneuploidy (Patwardhan et al., 2018). To explore the relationship between STIL expression and aneuploidy in cancers, we used a recently defined aneuploidy score (Taylor et al., 2018). We observed a positive correlation in pan-cancer between STIL mRNA expression and aneuploidy score (Supplementary Figure S5C). To control the STIL CNV as a confounding variable in aneuploid tumors, we conducted the same analysis by only utilizing diploid samples, which were identified with no copy number alteration. Intriguingly, we observed a stronger correlation between STIL mRNA expression and aneuploidy score in diploid pan-cancer samples (Supplementary Figure S5D), suggesting that genomic instability was affected by over-expressed STIL, but not a by-product of STIL CNV.

### Molecular Mechanisms and Biological Functions of STIL

**FIGURE 3:**
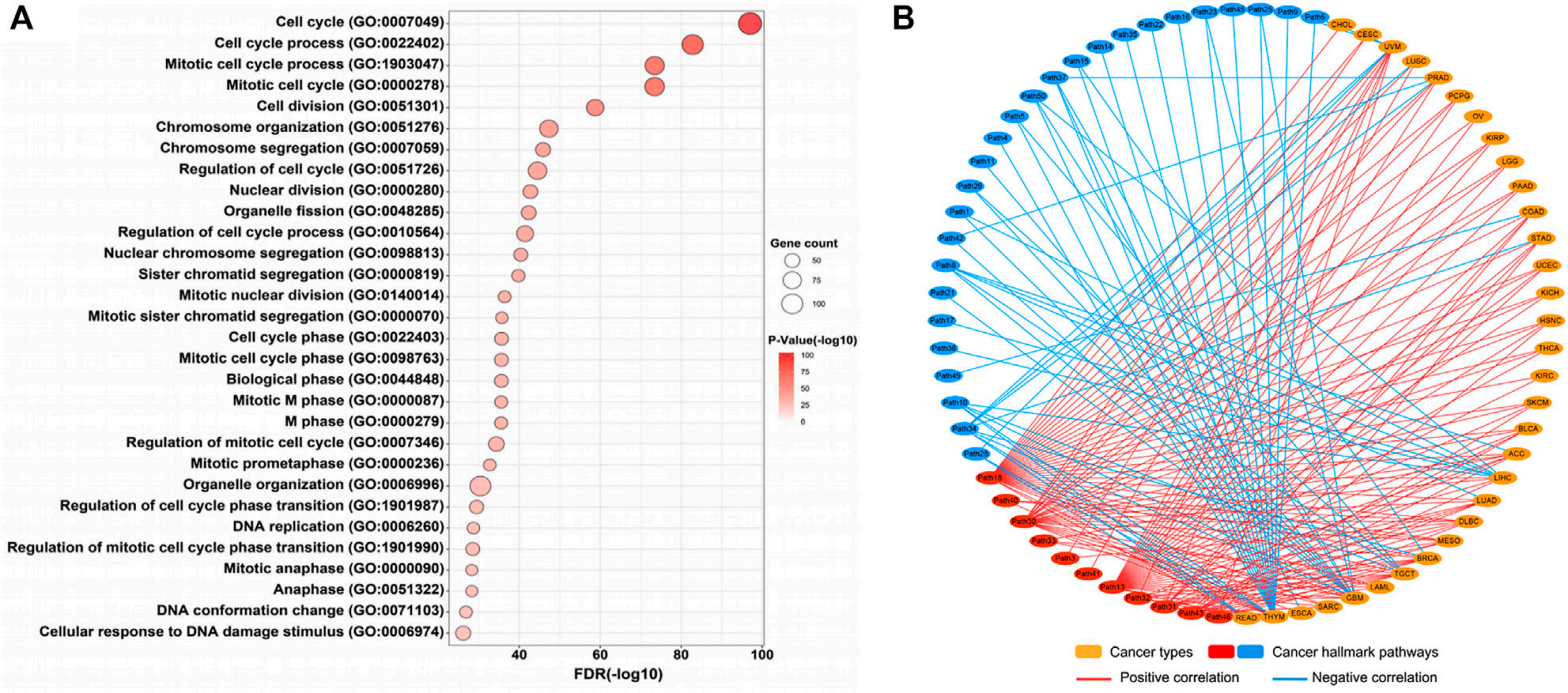
Molecular mechanisms of STIL. **(A)** Genetic ontology enrichment analysis of STIL co-expression genes reveals the potential biological progression of STIL. The bubble chart shows the top 30 biological progressions. The size of the bubble showed gene counts involved in biological progressions. The shade of color showed a *p*-value, the redder the color, the smaller the *p*-value. **(B)** The network diagram showed the correlation between STIL and hallmark-related cancer pathways across 33 cancer types. The red showed a positive correlation, and the blue showed a negative correlation. The details of the pathway name are shown in Supplementary Table S7.

Although many studies indicated that STIL dysregulation directly affected genomic instability and cell cycle of tumors (Holland and Cleveland, 2009; David et al., 2014; Patwardhan et al., 2018), the potential molecular mechanisms and biological functions of STIL were still ambiguous in pan-cancer. To explore the biological function of STIL, we filtered 175 co-expression genes of STIL (Pearson Correlation Coefficient ≥ 0.6) in pan-cancer levels using the GEPIA website (Supplementary Table S4). Then we implemented GO functional enrichment analysis and visualized the top 30 biological processes (Figure 3A; Supplementary Table S5). The results showed that STIL is mainly involved in the cell cycle progression. Meanwhile, we found STIL was positively or negatively correlated with multiple cancer hallmark-related pathways across 33 cancer types by Gene Set Variation Analysis (Supplementary Figure S6; Supplementary Table S6). We regarded PCC > 0.5 and PCC < −0.4 as significant correlations and further visualized the correlation characterization by a network diagram (Figure 3B). Interestingly, we found STIL showed a consistently positive correlation with three pathways across 33 cancer types, including mitotic spindle (Figure 3B, Path 30), G2M checkpoint (Figure 3B, Path 18), and E2F targets pathways (Figure 3B, Path 13).

**FIGURE 4:**
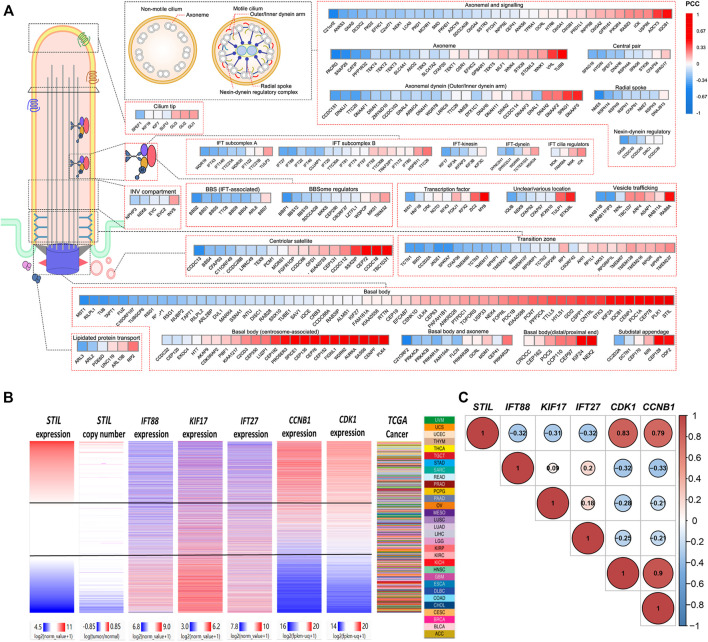
Correlation of STIL and cilia, cell cycle proteins. **(A)** A correlation heatmap landscape between STIL expression and cilia proteins representing different structures or functions of cilia. The positive correlations are colored by red, and the negative correlations are colored by blue. **(B)** Comparison of STIL mRNA expression, STIL copy number, IFT88 mRNA expression, KIF17 mRNA expression, IFT27 mRNA expression, CCNB1 mRNA expression, and CDK1 mRNA expression in all TCGA primary tumors with overlapping genomics data. Data were retrieved from UCSC Xena. **(C)** The correlation diagrams show the co-expression situation among STIL, IFT88, KIF17, IFT27, CDK1, and CCNB1 in pan-cancer. The positive correlations are colored by red, and the negative correlations are colored by blue. The size of the point represents the significance.

STIL is a ciliary-related gene, which has been found closely correlated with the cell cycle progression of multiple tumors (Ye et al., 2020; Ouyang et al., 2020). Primary cilia showed a decrease or loss in multiple cancer types (Seeley et al., 2009; Basten et al., 2013; Nobutani et al., 2014; Du et al., 2018; Qie et al., 2020). However, the role of STIL for primary cilia still needs to explore in the tumor. For this purpose, we divided the genes into several kinds based on the structure or functions of primary cilia (including basal body, transition, IFT complex, BBS protein, axoneme, etc.) (Reiter and Leroux, 2017), and conducted a correlation analysis (Figure 4A, Supplementary Table S7). We found STIL expression was negatively correlated with most cilia axoneme and IFT family genes. These genes were required for ciliogenesis, such as C21orf2, DCDC2, CATIP, CCDC151, TTC25, NME5, IFT27, IFT88, KIF17, IFT43, etc. (Hayes et al., 2007; Bontems et al., 2014; Jerber et al., 2014; Schueler et al., 2015; Suga et al., 2016; Milic et al., 2017; Rabinowicz et al., 2017b; Álvarez-Satta et al., 2017; Cho et al., 2020; Shohayeb et al., 2020) We also found that STIL expression showed a negative correlation with multiple BBS genes, such as BBS1, BSS2, BBS5, BBS12, etc. On the contrary, STIL expression was positively correlated with GLI proteins (Figure 4A). The GLI proteins were the main downstream effectors for SHH signaling (Cayuso et al., 2006). SHH signaling was considered to be associated with primary cilia and required STIL to activate (Scholey and Anderson, 2006; Goetz et al., 2009). Furthermore, STIL expression showed a highly positive correlation with multiple basal body (BB) genes, such as PLK1, PLK4, AURKA, WDR62, KIF2A, KIF24, CEP family proteins, which were involved in centrosome duplication or primary cilia degradation (Lee et al., 2012; Kumar et al., 2013; Kim et al., 2015; Hori et al., 2016; Zhang et al., 2019). We further selected several pivotal genes which participate in the cilia formation (including IFT88, KIF17, and IFT27) and cell cycle (including CDK1 and CCNB1) to conduct subsequent analysis. A heatmap and correlation diagram were visualized using the UCSC Xena Browser Heat Maps and R-project (Figures 4B,C). We observed that STIL expression showed a highly positive correlation with CDK1 and CCNB1, and these two cycle proteins also represented a negative correlation with IFT88, KIF17, and IFT27. These results implied that STIL was involved in regulating primary cilia formation and cell cycle progression.

### STIL Regulated Cell Cycle Progression in Cilia-dependent Manner

**FIGURE 5:**
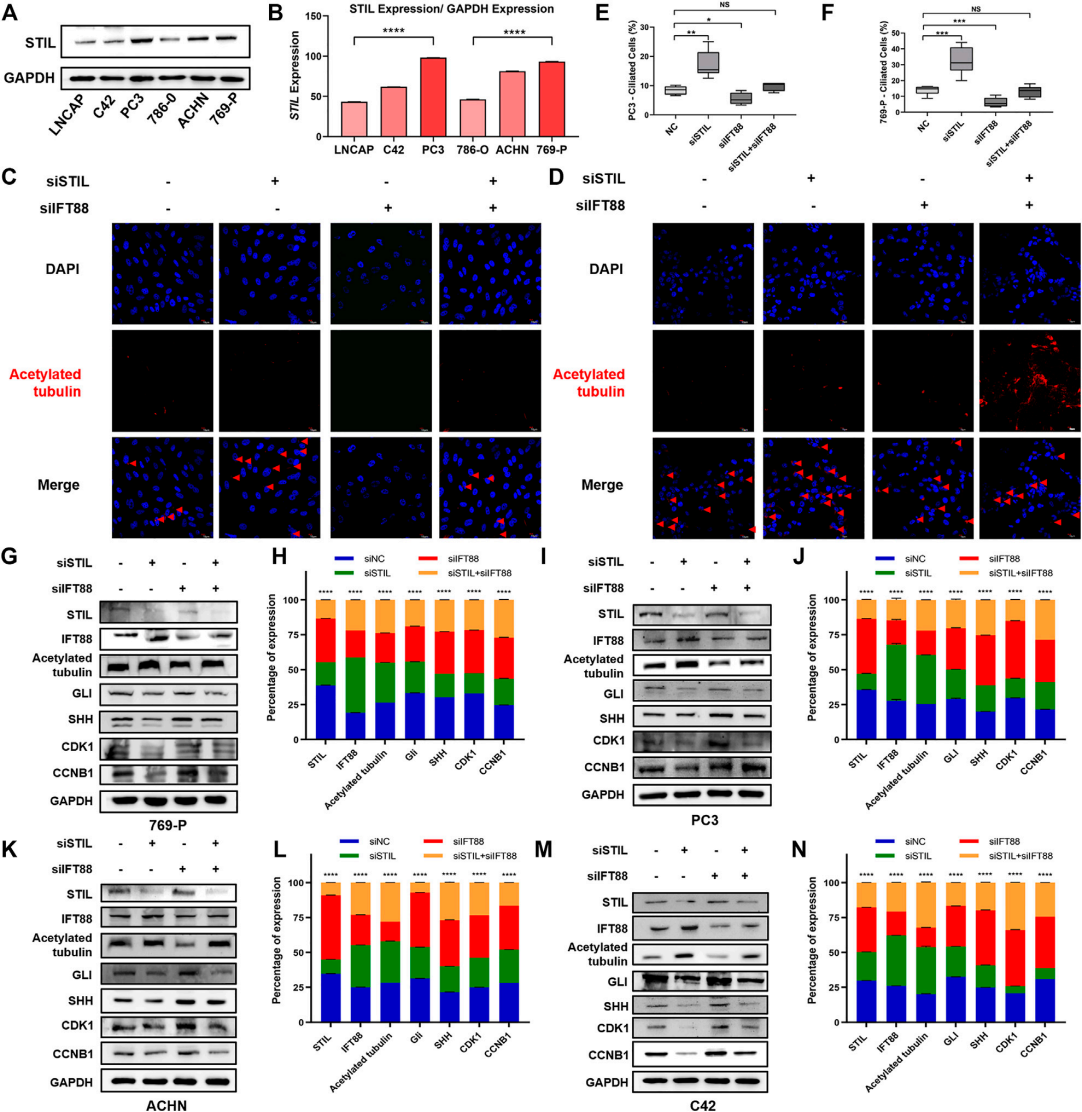
STIL regulated cell cycle progression in a cilia-dependent manner in prostate and kidney cancer cells. **(A,B)** Western blot analysis for the protein expression of STIL in the prostate (including LNCaP, C42, and PC3) and kidney (including 786-O, ACHN, and 769-P) cancer cells. **(C,D)** Control cells (including PC3 and 769-P) and cells stably transfected with nontarget shRNA, STIL shRNA, IFT88 shRNA, and STIL-IFT88 shRNA were serum-starved for 48 h fixed and stained with antibodies against Ace-tubulin (red) and DAPI (blue) for immunofluorescence analysis. The scale bar indicates 10 μm. **(E,F)** The percentages of ciliated cells in the cells stably transfected with nontarget siRNA, STIL siRNA, IFT88 siRNA, and STIL-IFT88 siRNA groups **(G,I,K,M)** Western blot analysis for the protein expression (including STIL, IFT88, Ace-tubulin, CDK1, CCNB1, GLI, and SHH) in the control group cells (including C42, PC3, 769-P, and ACHN) and cells stably transfected with nontarget shRNA, STIL shRNA, IFT88 shRNA, and STIL-IFT88 shRNA groups **(H,J,L,N)** Percentage of protein expression in nontarget shRNA, STIL shRNA, IFT88 shRNA, and STIL-IFT88 shRNA groups by Western blot in C42, PC3, 769-P, ACHN cell lines (*n* = 3, mean ± SEM). ****p-value < 0.0001, multiple *t*-test.

To explore the effect of STIL on primary cilia formation and cell cycle in the tumor cell, we, respectively, selected 3 cell lines representing different malignancy degrees of prostate cancer (LNCaP, C42, and PC3) and kidney cancer (786-O, ACHN, and 769-P) to conduct subsequent research. We found that the expression of STIL-encoding protein was increased with the advanced malignancy degree of cell lines (Figures 5A,B). Due to STIL showing the highest protein expression in 769-P and PC3, we selected these 2 cells to conduct subsequent analysis. We used acetylated tubulin as a cilia marker to observe the primary cilia frequency of prostate and kidney cancer cell lines. We found that STIL knockdown could increase the positive percentage of primary cilia in PC3 and 769-P cells (Figures 5C–F). However, IFT88 silencing decreased the percentage of cells with primary cilia. Interestingly, in STIL/IFT88-knockdown group, we observed that the percentage of cells with primary cilia was increased compared with the IFT88-knockdown group, indicating STIL silencing might reverse the outcome of IFT88 silencing on primary cilia. Moreover, we further performed to examine the cilia-related proteins (including IFT88 and acetylated-tubulin), cycle proteins (including CDK1 and CCNB1), and SHH pathway proteins (including SHH and GLI) levels in PC3 and 769-P cells. We found that IFT88 and acetylated-tubulin expression levels were increased in STIL depletion cells (Figures 5G,H), but GLI, SHH, CCNB1, and CDK1 expression showed a significant decrease. Conversely, in IFT88 knockdown cells, acetylated-tubulin expression was down-regulated, the GLI, SHH, CDK1, and CCNB1 expressions were slightly elevated, whereas STIL expression was not affected. Furthermore, the expression alterations in SHH signaling and cell cycle proteins were reversed by STIL silencing in IFT88 depletion cells. We then performed the same treatments in the ACHN and C42 cells and observed similar results (Figures 5I–N). These results elucidated that STIL regulated cell cycle progression through primary cilia in prostate and kidney cancer cells.

## Discussion

Primary cilia must retract into the cell before cells enter into mitosis, indicated that primary cilia could prevent cells from occurring uncontrolled proliferation (Jones and Chen, 2008). The energy supply unit of primary cilia is centriole—a key structural element of the centrosome (Vulprecht et al., 2012). Cilia protein STIL is an essential component of the centriole replication machinery and primary cilia formation. STIL (−/−) mouse embryos do not contain centrioles or primary cilia—a phenotype that can be reversed by restoration of STIL expression (David et al., 2014). In multiple cancers, STIL expression has been revealed up-regulation (Goto et al., 2013; Rabinowicz et al., 2017a; Wu et al., 2019), and the number of primary cilia decrease or loss (Seeley et al., 2009; Basten et al., 2013; Nobutani et al., 2014; Qie et al., 2020). Our previous study also revealed that STIL was up-regulated, and the positive percentage of primary cilia is reduced in bladder cancer (Du et al., 2018). However, the function and mechanism of STIL in pan-cancer and how STIL regulates primary cilia in tumors still need to be explore.

In this study, we unmasked for the first time that STIL expression was up-regulated in most tumor tissues than that in the adjacent normal tissues. High STIL expression was accompanied by advanced pathological stage and malignancy degree of cancers, revealing STIL act as an oncogene can promote cancer progression (Ramaswamy et al., 2003). A previous study also indicated that STIL knockdown suppresses the progression of gastric cancers (Wang et al., 2019). In addition, STIL could promote the proliferation, migration, and invasion of the nasopharyngeal (Ouyang et al., 2020). These findings demonstrated that the potential of targeting STIL in cancer therapy. Furthermore, high STIL expression patients showed a worse prognosis in most cancer types. However, in READ and THYM, high STIL expression acted as a protective factor, indicating distinct heterogeneity prognostic values of STIL in different cancers.

A previous study revealed that STIL mutation could result in familial microcephaly (Mouden et al., 2015). In pan-cancer, STIL mutation characterization plays a weaker role in regulating STIL expression in cancers except for BLCA. Interestingly, the STIL DNA methylation level showed significant down-regulation in multiple cancer types. However, in HNSC, STIL methylation was positively correlated with STIL mRNA expression. The opinion cannot explain that DNA methylation silences gene expression (Neri et al., 2017). Therefore, the role of STIL methylation in regulating its expression still needs further validation. It is generally accepted that somatic CNV is highly related to the development and progression of numerous cancers by impacting gene expression levels (Zhao et al., 2014; Kwak et al., 2015; SamulinErdem et al., 2017; Yang et al., 2017; Zhou et al., 2017; Gut et al., 2018). A pan-cancer analysis revealed that copy number displays a positive linear influence on gene expression for the majority of genes, genetic variation directly generated a transcriptional gene level (Shao et al., 2019). Our research also discovered a positive correlation between STIL expression and copy number, indicating that copy number was one of the factors affecting STIL expression. Of course, though the impact of mutation and DNA methylation in STIL expression was not noticeable, we still cannot ignore their existence because the role of these epigenetic characterizations was different in various cancer types.

Cells begin the cell cycle with exactly one centrosome, and the duplication of centrioles is constrained such that it occurs only once per cell cycle and at a specific site in the cell (Nigg and Stearns, 2011). STIL and its interactors are central to this process, ensuring the fidelity of this unique duplication, thereby minimizing chromosome instability (Funk et al., 2016). Abnormally high expression of STIL in differentiated tissues triggered centrosomal amplification and accelerated cell cycle progression (Vulprecht et al., 2012). Both overduplication and lack of duplication of centrosomes lead to abnormal mitotic spindle assembly and consequently increase the chances of abnormal chromosomal segregation and aneuploidy (Holland and Cleveland, 2009). Aneuploidy was defined as a somatic copy number alternations class correlated with chromosomal instability (Taylor et al., 2018). Our results showed that STIL expression was positively correlated with aneuploidy score in pan-cancer. Interestingly, the correlation between STIL and aneuploidy was retained in tumors with diploid samples, suggesting that increased STIL expression promotes centriole duplication and aneuploidy formation, rather than just representing a by-product of STIL CNV. The formation of aneuploidy could provide an evolving genome to help the cells adapt to the changing environment of cancer and escape normal checkpoints (Patwardhan et al., 2018), revealing the cancer promotion function of STIL over-expression from the side.

The Gene Ontology (GO) is a community-based bioinformatics resource that offers information about gene product function using ontologies to represent biological knowledge (Gene Ontology Consortium, 2015). GO enrichment analysis revealed that STIL was involved in processes associated with the cell cycle, consistent with the previous study (Arquint et al., 2012; Vulprecht et al., 2012). Besides, STIL expression was positively correlated with the Mitotic spindle, G2M checkpoint, and E2F targets pathways across 33 cancer types, suggesting that the molecular mechanisms of STIL in cancers were stable and did not change with cancer type. STIL upregulation was associated with a high histopathological mitotic index in tumors and presumably affected mitotic spindles formation (Lattao et al., 2017). A previous study also reported that downregulation of STIL prevents G2-M transition, slows down cell cycle progression (Erez et al., 2008). Moreover, E2F knockdown downregulated STIL expression and regulated G2/M transition (Erez et al., 2004). Therefore, STIL expression alteration could regulate the cell cycle and eventually promote the occurrence of cancer.

Evidence also was demonstrated that the primary cilia are involved in cell cycle regulation (Goto et al., 2013; Lattao et al., 2017). The resorption of the primary cilia during the cell cycle allows the centrosome to detach from the basal body and become the centrosome and the mitotic spindle. They can then continue to carry out their role in the cell cycle (Goto et al., 2013). Our results indicated that STIL expression was negatively correlated with multiple cilia axoneme, IFT family, and BBS protein. Cilia axoneme and IFT family are required in ciliogenesis (Pazour et al., 2000; Robert et al., 2007; Dishinger et al., 2010; Eguether et al., 2014; Reiter and Leroux, 2017). Bardet-Biedl syndrome (BBS) is a rare autosomal recessive ciliopathy. BBS genes encode proteins that localize to the cilia and basal body and are involved in cilia biogenesis and function (Forsythe and Beales, 2013; Álvarez-Satta et al., 2017). Furthermore, STIL was positively related to most centrosome-associated genes. These genes promote centrosome duplication and participate in cell cycle progressions. In pan-cancer levels, STIL showed consistent expression alteration with CDK1 and CCNB1. CDK1/CCNB1 were crucial cell cycle proteins, regulating mitochondrial bioenergetics in cell cycle progression and tumor resistance (Xie et al., 2019). Previous research also indicated that increasing STIL could promote CDK1/CCNB1 activity and indirectly participate in CCNB1 dependent proliferation in tumor cells (Erez et al., 2007). These results provided potential information that STIL expression could repress cilia formation and regulate the cell cycle.

To understand the effect of STIL on primary cilia and cell cycle, we utilized prostate and kidney cancer cell lines to conduct subsequent validation. Studies have shown a close relationship between the development of these two cancer types and primary cilia loss (Dere et al., 2015; Qie et al., 2020). In the cell lines representing different malignancy degrees of these two cancer types, STIL protein expression in PC3 and 769-P showed the highest levels. Considering that STIL disturbance could observe an obvious phenotype, we ultimately selected PC3 and 769-P to conduct the subsequent experiment. We found the effects of STIL on primary cilia and cell cycle proteins could be reversed by IFT88. Meanwhile, STIL depletion could decrease the SHH pathway activity, an essential signaling pathway to the presence of primary cilium (Goetz et al., 2009). These results verified that STIL over-expression could repress the formation of primary cilia and further promote the cell cycle progression of prostate and kidney cancer cells.

In summary, our results revealed the importance of STIL in pan-cancer. Up-regulated STIL was associated with poor prognosis and recurrence of cancer, promoting genomic instability and aneuploidy formation. STIL could regulate the cancer cell cycle through primary cilia. All in all, STIL was an crucial molecular marker and might be a novel targeted therapy for cancer.

## Materials and Methods

### Collection and Progression of Public Data

The RNA-seq expression data of the TCGA database were obtained from the UCSC Xena (https://xena.ucsc.edu/). We totally analyzed 33 different cancer types from the TCGA datasets, including ACC, adrenocortical carcinoma; KIRC, kidney renal clear cell carcinoma; KICH, kidney chromophobe; KIRP, kidney renal papillary cell carcinoma; GBM, glioblastoma multiforme; LGG, brain lower grade glioma; BRCA, breast cancer; LUAD, lung adenocarcinoma; LUSC, lung squamous cell carcinoma; COAD, colon adenocarcinoma; DLBC, lymphoid neoplasm diffuse large b-cell lymphoma; PAAD, pancreatic adenocarcinoma; ESCA, esophageal carcinoma; TGCT, testicular germ cell tumors; READ, rectum adenocarcinoma; UCS, uterine carcinosarcoma; UCEC, uterine corpus endometrial carcinoma; OV, ovarian serous cystadenocarcinoma; HNSC, head and neck squamous carcinoma; THCA, thyroid carcinoma; SKCM, skin cutaneous melanoma; BLCA, bladder urothelial carcinoma; LIHC, liver hepatocellular carcinoma; PRAD, prostate adenocarcinoma; STAD, stomach adenocarcinoma; CESC, cervical squamous cell carcinoma and endocervical adenocarcinoma; PCPG, pheochromocytoma and paraganglioma; SARC, sarcoma; MESO, mesothelioma; UVM, uveal melanoma; LAML, acute myeloid leukemia; THYM, thymoma; CHOL, cholangiocarcinoma.

All the TCGA RNA-seq expression value was normalized and transformed to CPM unit by utilizing edgR R package to allow for subsequent analyses. The DNA methylation data and clinical information across 33 cancer types were also downloaded from the UCSC Xena. The copy number variation (CNV) data based on the TCGA across 33 cancer types were acquired from the cBioPortal website (https://www.cbioportal.org/) and UCSC Xena. cBioPortal website is an open-access resource for interactive exploration of multidimensional cancer genomic data sets (Cerami et al., 2012). The amplification or missing thresholds of CNV were assessed using the GISTIC2 method (Mermel et al., 2011). The somatic mutation data were obtained from the public website (https://pancanatlas.xenahubs.net/download/) containing >9,000 samples to calculate the mutation frequency. Clinical information of TCGA cancer patients was also downloaded from UCSC Xena, including survival data, histological grade, ages, and pathological stage.

Meanwhile, we downloaded the genome-wide copy number variation and RNA expression of 1,100 cancer cell lines across 22 cancer types from the Cancer Cell Line Encyclopedia (CCLE) database (https://portals.broadinstitute.org/ccle) and calculated the correlation between STIL CNV and STIL expression, aiming to validate the results analyzed from the TCGA database. The amplification or deletion threshold of CCLE CNV: CNV ≥ 0.4: Amplification; 0.2 ≤ CNV < 0.4: Gain; −0.2 < CNV < 0.2: diploid; −0.4 < CNV ≤ −0.2: Shallow deletion; CNV ≤ −0.4: Deep deletion.

To verify the results of expression alteration analyzed from the TCGA database, we downloaded mRNA expression data of 17 cancer types from the GEO datasets (https://www.ncbi.nlm.nih.gov/gds). The RNA expression was transformed to log_2_ (counts + 1) unit and normalized by the limma package based on R software.

### Expression, CNV, and DNA Methylation Analysis of STIL

The Gene expression profiling interactive analysis (GEPIA) was used to depict the expression pattern of STIL in human body organs. For the TCGA dataset, we firstly carried out an unpaired *t*-test analysis to compare the expression difference of STIL in different cancer types with their corresponding normal samples. Fifteen cancer types containing normal samples from the GEO database were selected To verify the STIL expression alteration. Meanwhile, 23 TCGA cancer types were selected to conduct differential methylation analysis. For each cancer type, we performed a differential expression or methylation analysis using the limma R-package downloaded from Bioconductor (https://www.bioconductor.org/) (Ritchie et al., 2015). We used Wilcox’s rank-sum test to identify different expression genes and the Benjamini Hochberg method to adjust the *p*-value. The adjusted *p*-value <0.05 and absolute log_2_ fold change >0.5 were regarded as significant. The differentially methylated regions were filtered using the Beta value of DNA methylation (change greater than 0.2 between tumor and normal pairs) and false discover rate (FDR) < 0.05 (based on the Wilcoxon rank‐sum statistical nonparametric test and Benjamini‐Hochberg method). For the correlation analysis, |Pearson *r*| > 0.3 or |Spearman *r*| > 0.3 and *p* value <0.05 was regarded as significant. The *p*-values of the significant differences are identified with asterisks. *p-value < 0.05; **p-value < 0.01; ***p-value < 0.001; ****p-value < 0.0001; NS, No Significance.

### Survival Analysis of Pan-Cancer

The univariate Cox analysis was conducted to assess the correlation between STIL expression and patient survival status from the TCGA database. The hazard regression model was utilized to assess the prediction performance of STIL. Moreover, we plotted with the Kaplan-Meier survival curves using the GEPIA website (Tang et al., 2017) and used the log-rank test to estimate the survival difference comparing the high STIL expression cohort with the low STIL expression cohort. In addition, the PrognoScan website (http://dna00.bio.kyutech.ac.jp/PrognoScan-cgi/PrognoScan.cgi), which collected the survival data of various cancer type patients (Mizuno et al., 2009), was also used to assess the correlation between STIL and patient survival.

### Mechanism and Biological Process Analysis

In the GEPIA website, we selected the similar genes analysis and added all 33 cancers to analyze the co-expression genes of STIL. The top 200 similar genes were presented in the interface. We regarded the PCC ≥ 0.6 as the highly correlated genes of STIL. 175 co-expression genes of STIL were subsequently determined across 33 cancer types and ultimately submitted to the public website (http://geneontology.org/) to conduct the GO analysis. Then, we downloaded gene sets of 50 hallmark cancer pathways from the GSEA website (https://www.gsea-msigdb.org/gsea/index.jsp) and calculated the pathway activity scores by Gene Set Variation Analysis (GSVA) (Hänzelmann et al., 2013) using genome-wide RNA-seq expression from the TCGA tumor data. We matched the STIL expression and 50 pathways activity scores in the same tumor sample. Then the Pearson Correlation Coefficient (PCC) between STIL expression and pathway activity scores was calculated in 33 cancer types, respectively, using *Hmisc* R software and visualized by the Cytoscape software (Shannon et al., 2003). UCSC Xena Browser Heat Maps were depicted by utilizing the UCSC Xena website (https://xenabrowser.net/).

### Cell Culture and siRNA Molecular Transfection

Human kidney cancer cell lines (786-O, 769-P, and ACHN) and prostate cancer cell lines (LNCaP, C42, and PC3) were purchased from the Cell Resource Center Affiliated to the Chinese Academy of Medical Sciences and cultured in RPMI-1640 medium (HyClone, Logan, UT, United States) supplemented with 10% fetal bovine serum (HyClone, Logan, UT, United States) and 5% penicillin-streptomycin (100 units/ml) in a humidified atmosphere incubator of 5% CO_2_ at 37°C. The cell culture medium was changed every 2–3 days depending on the cell density. For routine channel, cells were split at a ratio of 1:2–3 when they reach 80%–90% gather. STIL and IFT88 were silenced using siRNA oligonucleotides. The positive sequence of STIL was 5′-GCA​GUG​AUC​UCU​GGA​UUA​ATT-3′, the reverse sequence of STIL was 5′-UUA​AUC​CAG​AGA​UCA​CUG​CTT-3′. The positive sequence of IFT88 was 5′-GCC​AUU​AAA​UUC​UAC​CGA​ATT-3′, the reverse sequence of IFT88 was 5′-UUC​GGU​AGA​AUU​UAA​UGG​CTT-3′.

### Protein Extraction and Western Analysis

After transfecting for 48 h, cultured cells were gained and lyzed in six well plates by using total protein extraction reagent added 10% PMSF, then centrifuged, taken the supernatant. The concentrations of total proteins in the cell lysates were determined by the BCA assay. Individual cell lysates were separated by sodium dodecyl sulfate polyacrylamide gel electrophoresis (SDS-PAGE) on 10% gels and transferred electronically onto membranes made of polyvinylidene difluoride. The membranes were treated with 5% fat-free dry milk diluted by TBST buffer, and then incubated with antibodies against IFT88, STIL, Acetylated-Tubulin, SHH, CDK1, CCNB1, GLI, and GAPDH at 4°C overnight, respectively. The bound antibodies were detected with HRP‐conjugated secondary antibodies and visualized using Immobilon™ western chemiluminescent HRP Substrate.

### Immunofluorescence

Cells of each group (including normal control, STIL silencing, IFT88 silencing, and STIL-IFT88 silencing groups) were, respectively, grown on three glass coverslips (15 mm diameter) and fixed in 4% paraformaldehyde (PFA) for 10 min at room temperature. The coverslips were washed in PBS three times for 5 min. Then cells were permeabilized with 0.5% TritonX-100 for 5 min and then briefly blocked with 1% bovine serum albumin (BSA) solution in PBS for 10–15 min. The cells were incubated with ciliary marker antibodies against acetylated tubulin (1:1,000 dilution, Sigma-Aldrich) in blocking buffer at 4°C overnight. After incubating with TRITC-labeled anti-mouse secondary antibody (1:200 dilution, Southern Biotech) for 2 h, the nuclei were stained with 4’,6-diamino-2-phenylindole (DAPI, Solarbio). Primary cilia were observed in randomly selected ×400 microscopic fields (2–5 fields in each glass coverslips).

### Statistical Analyses

Unpaired *t*-test analysis was carried out to compare the differenced of two groups. One-way analysis of variance (ANOVA) was carried out to compare the differences of three and more groups. The log-rank test was used to estimate the prognosis of patients. Meanwhile, Pearson and Spearman were common methods to assess the correlation between the two groups (Bishara and Hittner, 2012). For all analyses, the difference was considered statistically significant when a *p*-value < 0.05.

## Data Availability

The original contributions presented in the study are included in the article/Supplementary Material, further inquiries can be directed to the corresponding authors.
