# Supplementary material for: STIL Acts as an Oncogenetic Driver in a Primary Cilia-Dependent Manner in Human Cancer
Source: Front Cell Dev Biol. 2022 Jan 26;10:804419. doi: 10.3389/fcell.2022.804419 (PMC8826476; doi:10.3389/fcell.2022.804419)
Supplement: Supplementary file 1 [file Image1.PDF]

STIL acts as an oncogenetic driver in a primary cilia-dependent manner in human cancer

Jingxian Li[1,3], Zikun Yang[1,3], Yuanjiong Qi[1], Xun Liu[1], Yang Liu[1], Xinyu Gao[2], Shuai Li[2], Jianqiang Zhu[1], Changwen Zhang[1], E Du[1*], Zhihong Zhang[1*]

[1] Tianjin Institute of Urology, The Second Hospital of Tianjin Medical University.
[2] Department of Graduate School Tianjin Medical university, Tianjin Medical University.
[3] Jingxian Li, and Zikun Yang contribute equal to this article.
[*]Corresponding author: E Du, Email: duedoc@tmu.edu.cn, Zhihong Zhang, Email: zhangzhihongtianj@163.com.

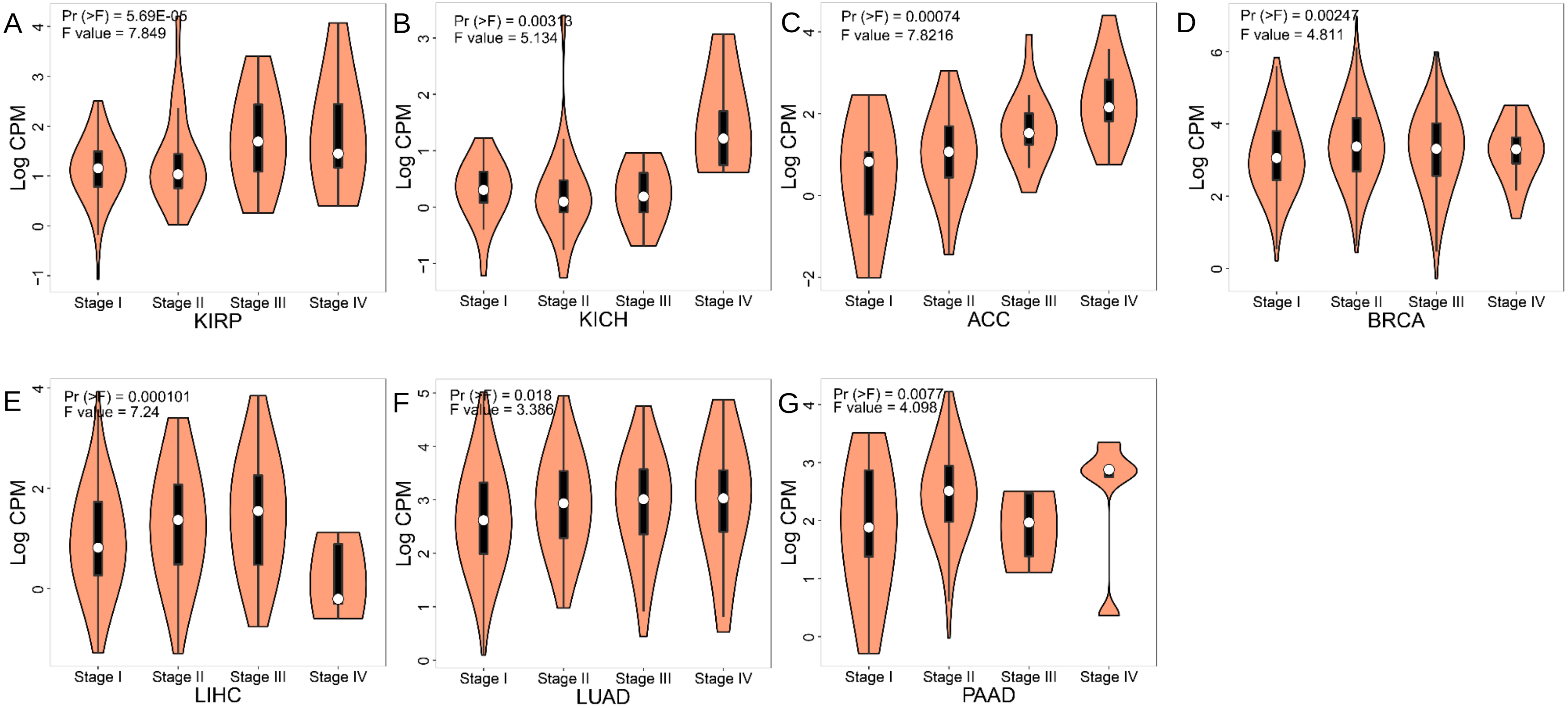

Figure S1 The violin diagram depicted the STIL mRNA expression in the different pathological stages in KIRP, KICH, ACC, BRCA, LIHC, LUAD, and PAAD. One-way analysis of variance (ANOVA) was carried out to compare the differences of more groups.

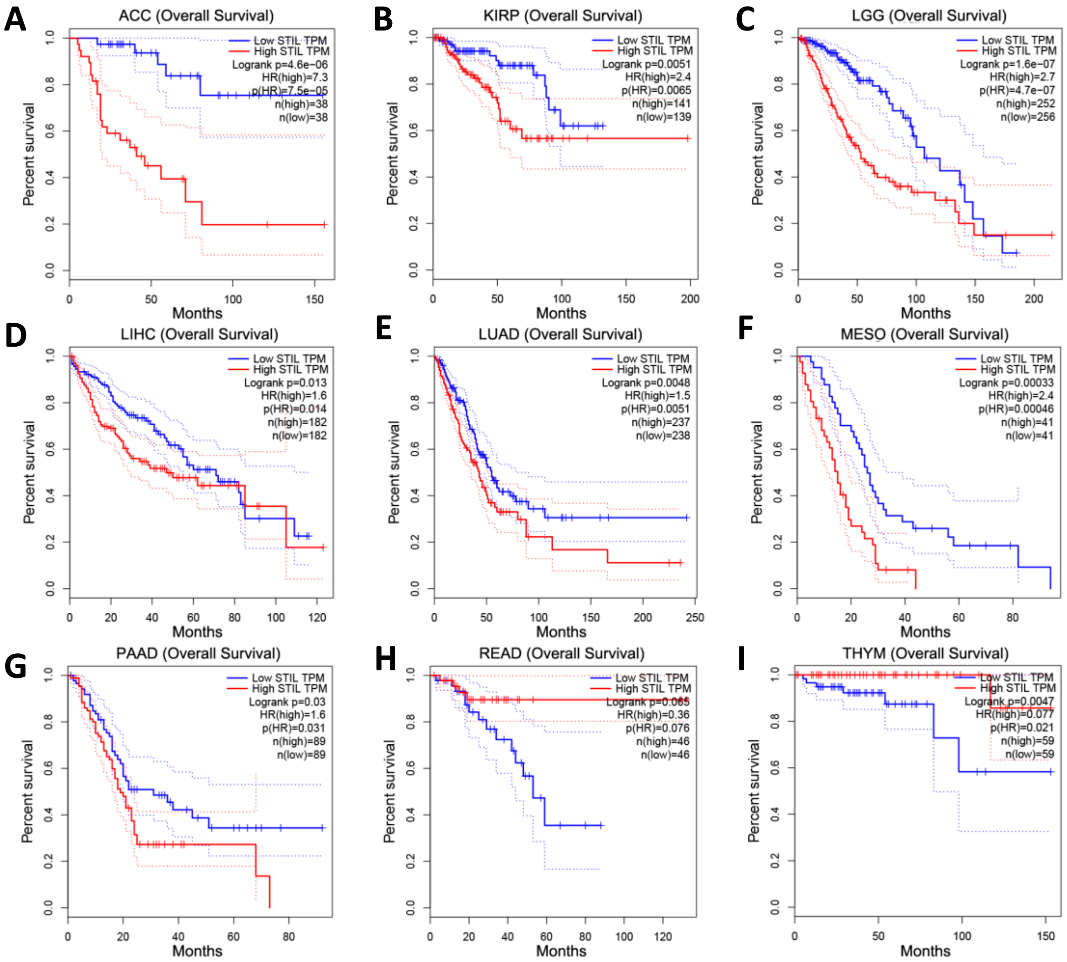

Figure S2 Kaplan-Meier overall survival curves of patients grouped by STIL expression in individual cancer from the TCGA datasets. The P-values calculated by the log-rank test are shown.

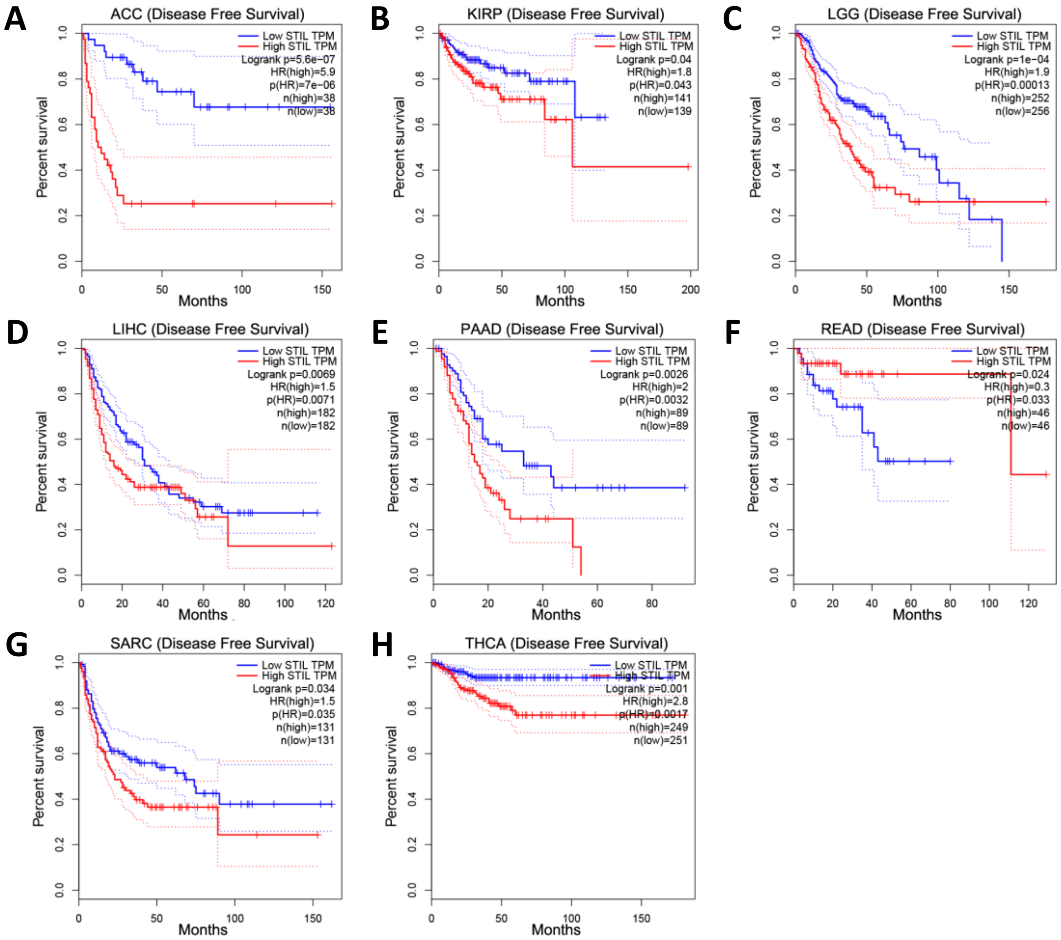

Figure S3 Kaplan-Meier disease-free survival curves of patients grouped by STIL expression in individual cancer from the TCGA datasets. The P-values calculated by the log-rank test are shown.

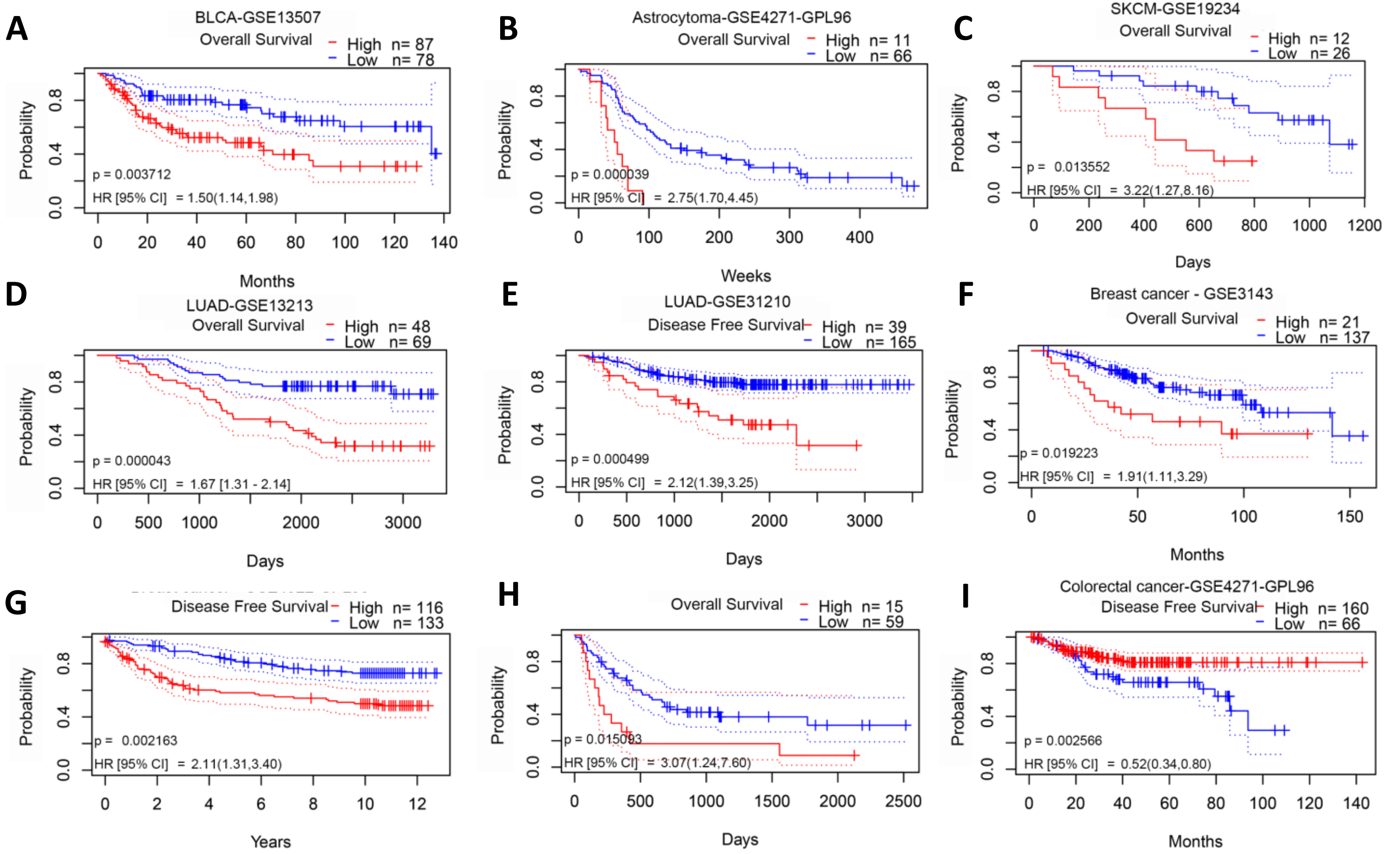

Figure S4 Kaplan-Meier survival curves of patients grouped by STIL expression in individual cancer from the GEO datasets. The P-values calculated by the log-rank test are shown.

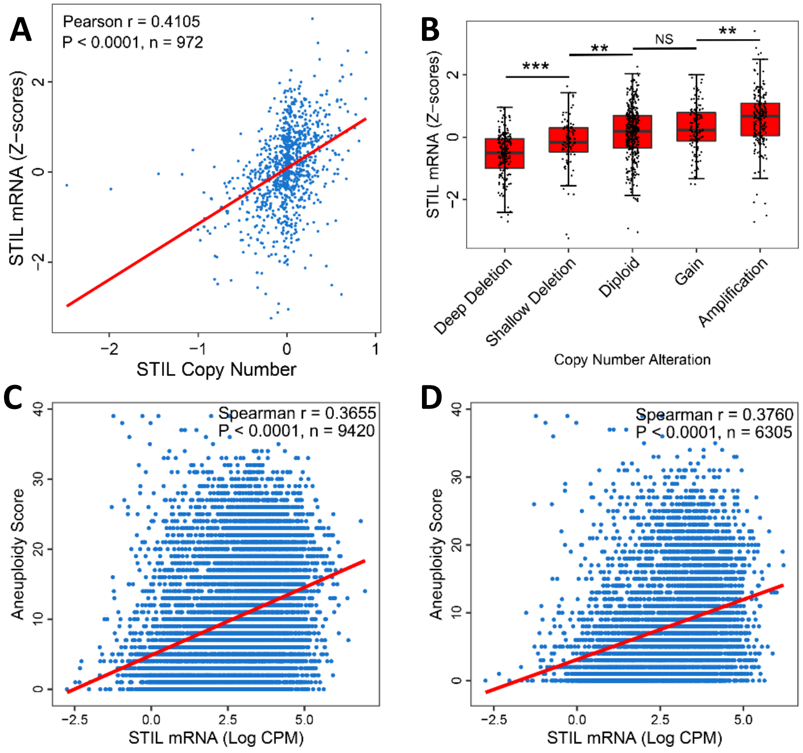

Figure S5 (A) Correlation between STIL mRNA expression and copy number variation in cancer cell lines. P-value, sample number, and Spearman r are shown in the box. (B) STIL mRNA expression in different copy number variation situations in cancer cell lines. Unpaired t-test analysis was carried out to compare the differenced of two groups.P-value and sample number are shown in the box. *, P-value < 0.05; **, P-value < 0.01; ***, P-value < 0.001; ****, P-value < 0.0001. (C) Correlation between STIL expression and aneuploidy scores in pan-cancer samples. (D) Correlation between STIL expression and aneuploidy scores in all diploid samples.

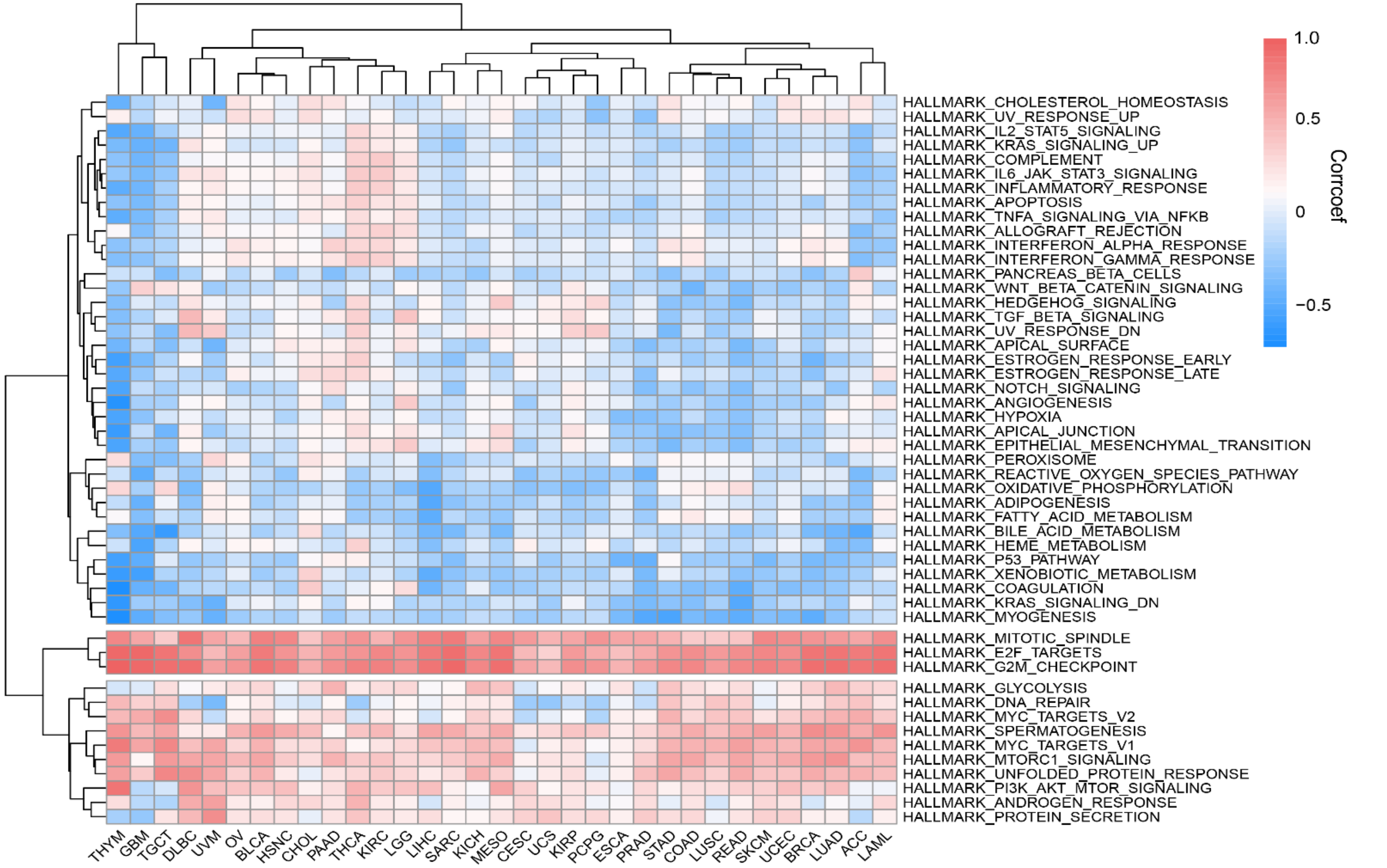

Figure S6 Heatmap showed the correlation between STIL expression and 50 hallmark-related cancer pathways. The red showed a positive correlation, and the blue showed a negative correlation.
